# Effects of Increasing Stimulated Area in Spatiotemporally Congruent Unisensory and Multisensory Conditions

**DOI:** 10.3390/brainsci11030343

**Published:** 2021-03-09

**Authors:** Chiara Martolini, Giulia Cappagli, Sabrina Signorini, Monica Gori

**Affiliations:** 1 Unit for Visually Impaired People, Center for Human Technologies, Istituto Italiano di Tecnologia, via Enrico Melen 83, 16152 Genoa, Italy; giulia.cappagli@iit.it (G.C.); monica.gori@iit.it (M.G.); 2 Center of Child Neuro-Ophthalmology, IRCCS Mondino Foundation, via Mondino 2, 27100 Pavia, Italy; sabrina.signorini@mondino.it

**Keywords:** unisensory and multisensory perception, increasing stimulated area, spatiotemporal coincidence, multisensory interaction, bodily stimulation

## Abstract

Research has shown that the ability to integrate complementary sensory inputs into a unique and coherent percept based on spatiotemporal coincidence can improve perceptual precision, namely multisensory integration. Despite the extensive research on multisensory integration, very little is known about the principal mechanisms responsible for the spatial interaction of multiple sensory stimuli. Furthermore, it is not clear whether the size of spatialized stimulation can affect unisensory and multisensory perception. The present study aims to unravel whether the stimulated area’s increase has a detrimental or beneficial effect on sensory threshold. Sixteen typical adults were asked to discriminate unimodal (visual, auditory, tactile), bimodal (audio-visual, audio-tactile, visuo-tactile) and trimodal (audio-visual-tactile) stimulation produced by one, two, three or four devices positioned on the forearm. Results related to unisensory conditions indicate that the increase of the stimulated area has a detrimental effect on auditory and tactile accuracy and visual reaction times, suggesting that the size of stimulated areas affects these perceptual stimulations. Concerning multisensory stimulation, our findings indicate that integrating auditory and tactile information improves sensory precision only when the stimulation area is augmented to four devices, suggesting that multisensory interaction is occurring for expanded spatial areas.

## 1. Introduction

Spatial representation arises from the reciprocal relationship between the perceiver and entities in the environment and the integration of multiple sources of sensory information from the surroundings [1]. The importance of visual feedback for spatial representation has been widely demonstrated [2,3,4,5]. For instance, vision facilitates the representation of space in allocentric coordinates [6,7,8], while the lack of visual input significantly interferes with the development of spatial competencies and alters allocentric perception of space [9,10,11,12]. Moreover, the presence of visual feedback can improve the spatial encoding of an event [13,14,15]. Nonetheless, it has been demonstrated that also the integration of distinct sensory information can enhance perceptual precision compared to unimodal stimulation when stimulation is spatially and temporally congruent [16,17]. For instance, the combination of visual-auditory [18], visual-tactile [19] and auditory-tactile [20] stimuli results in enhanced spatial and temporal discrimination abilities. Moreover, reaction times are shorter when multimodal rather than unimodal stimulation is provided [18,21]. Finally, it has been also shown that auditory and tactile information is strongly biased in the spatial domain when in conflict with simultaneous visual stimuli, suggesting that visual information dominates spatial perception [22,23,24,25,26,27].

Although much evidence indicates that multisensory information enhances perceptual abilities and improves detection and discrimination of stimuli compared to unimodal information, the mechanisms underpinning such perceptual benefits are still unknown. Several pieces of evidence indicated that temporal proximity affects multisensory integration. Stevenson and colleagues [28] showed that reaction times increased when visual and auditory stimuli were asynchronous and when synchronous visuo-auditory stimuli were located in the visual periphery. Temporal proximity influences the perception of multisensory stimuli according to the spatial region where the stimulation is provided. The space outside the body is divided into peripersonal (i.e., immediately around the body; [29,30,31,32] and extrapersonal (i.e., beyond the peripersonal region; [30]) spatial areas. According to such statement, Sambo and Foster [33] confirmed decreased reaction times to simultaneous visuo-haptic stimulation only when stimulation occurred in the peripersonal space. Several studies have also demonstrated that spatial proximity of unisensory stimulation promotes a statistically optimal sensory integration [34,35,36,37]. According to such results, it has been argued that typical adults’ performance in a size discrimination task depended on the spatial position of multiple visual and haptic stimuli, showing an improvement of performance only for spatially coincident stimulations. However, it is not clear whether the size of sensory stimulation can affect perceptual accuracy, specifically whether incrementing the overall sensory stimulated area with multiple spatially and temporally coincident stimuli would enhance or impoverish sensory discrimination. This effect is referred to the impact of the stimulated area’s size on the perceived intensity of a stimulation. Therefore, positive results would indicate that the more the surface area stimulated, the higher the intensity of the perceived stimulus. Similarly, spatial summation effects have been demonstrated at the perceptual level (e.g., for different visual stimuli [38,39,40], tactile stimuli [41,42,43] and pain stimuli [44]) and at the cortical level (e.g., in the visual cortex areas [45]), suggesting its potential role for several perceptual mechanisms. Moreover, several pieces of evidence have demonstrated that spatial summation effects might explain several psychophysical phenomena, e.g., contextual effects [46,47]. 

In the present study, we investigated the relationship between the size of the stimulated surface and sensory discrimination by assessing how the size of sensory stimulation influences perception in unimodal (visual, auditory, tactile) and multimodal (bimodal, trimodal) conditions. We asked participants to tap a sensitized surface with the right index finger as soon as they perceived unimodal (visual, auditory, or tactile) or multimodal (combination of unimodal stimuli) stimulations conveyed by multisensory units positioned on the left harm and then to verbally indicate the number of stimuli perceived, independently of the stimulus modality. We hypothesize that incrementing the stimulated area would decrease sensory threshold, thus increase sensory discrimination, both in unisensory and multisensory conditions. Moreover, due to the strong dominance of vision in perception, we expected that visual information would dominate multimodal stimulation. Specifically, vision relies on a reference system based on external landmarks and facilitates the representation of space in allocentric coordinates. Thus, we hypothesized that vision would promote the interaction of multiple stimuli conveyed on an increasing stimulated area of the body and enhance sensory accuracy more than modalities based on an egocentric perspective of space (e.g., touch). Indeed, we hypothesized that the absence of visual inputs would undermine an effective interaction between auditory and tactile stimulations, irrespective of the size of stimulated area. 

## 2. Materials and Methods

### 2.1. Participants

Sixteen sighted adults between 25 and 37 years of age (mean age: 29 ± 0.82 years, 10 females) were enrolled in the study. Participants were randomly recruited by Istituto Italiano di Tecnologia (Genoa, Italy), which provided them with monetary compensation for their participation. Participants belong to middle and upper-class Caucasian families living in a university town in Italy and none of them reported visual, auditory, musculoskeletal or neurological impairments. The study was approved by the local Ethics Committee (Comitato Etico Regione Liguria, Genoa, Italy; Prot. IIT_UVIP_COMP_2019 N. 02/2020, 4 July 2020), and participants gave written consent to the experimental protocol, following the Declaration of Helsinki. The sample size was calculated with the free software G * Power 3.1 (accessed on 4 July 2020, from www.psycho.uni-duesseldorf.de/abteilungen/aap/gpower3/), based on the following parameters:

- effect size d_z_: 1.18 (Cohen’s d = 1.09; see Schiatti et al., 2020 [48]);
- *α* err. prob. = 0.05;
- power (1 − *β* err. prob.) = 0.95.

### 2.2. Experimental Setup and Protocol

**Figure 1:**
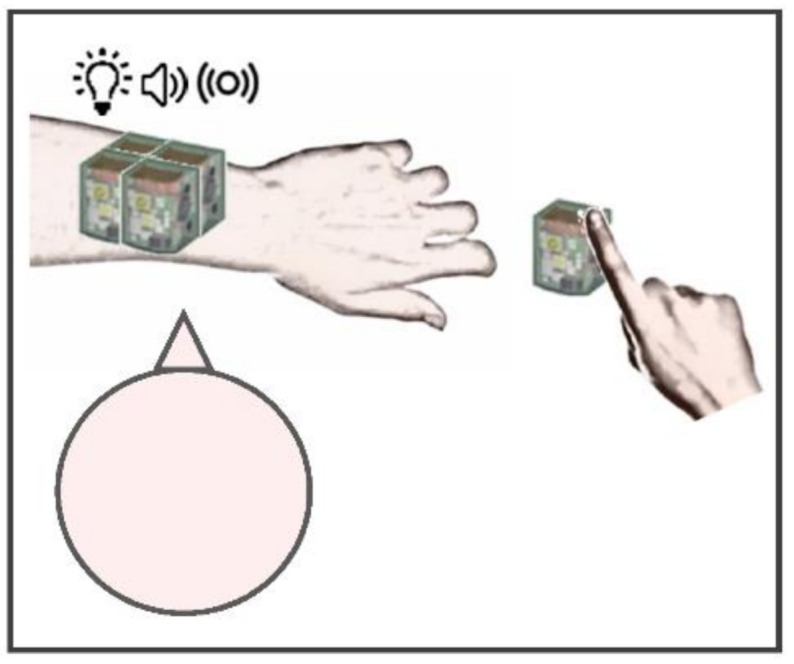
Experimental setup of the increasing stimulated area task. The system used for the increasing stimulated area task consisted in a technological system providing spatially and temporally coherent multisensory stimulation with real-time feedback from the user. Four units of the system were shaped in a 2 × 2 array and positioned on each participant’s left arm, with the head centrally aligned. Another unit was placed on the table, next to the right index finger, to record reaction times by tapping the upper sensitized surface. The dimensions of each unit were 2.5 × 2.5 cm^2^, for a total of 25 cm^2^ with four units.

The experiment was conducted in a dark room, where participants sat in front of a table. The experimental setup consisted of five multisensory units, part of a wearable, wireless system that provides spatially- and temporally-coherent multisensory stimulation with real-time feedback from the user. Specifically, the system is the TechARM, entirely designed and realized by Istituto Italiano di Tecnologia (Genoa, Italy) in collaboration with Center of Child Neuro-Ophthalmology, Istituto di Ricovero e Cura a Carattere Scientifico (IRCCS) Mondino Foundation (Pavia, Italy), with the main intent to assess and train impaired perceptual functions caused by vision loss from an early age. The system has been recently validated by Schiatti and colleagues (2020) to investigate to investigate spatial perception in interactive tasks. Each unit included embedded sensors and actuators to enable visual (red, green and blue (RGB) light-emitting diode (LED)), auditory (digital amplifier and speaker), and tactile (haptic moto-driver) interactions and a capacitive surface (capacitive sensor) on the device’s upper part to receive and record real-time inputs from the user (dimension of a single unit: 2.5 cm × 2.5 cm × 2.5 cm; dimension of upper sensitized area: 6.25 cm^2^). Four units were shaped in a 2 × 2 array and positioned on each participant’s left arm, which was centrally aligned with their head, while the fifth unit was placed on the table, next to the right index finger. The dimension of stimulation area was in the range from 6.25 cm^2^ (single unit: 2.5 × 2.5 cm^2^) to 25 cm^2^ (four units) (Figure 1). During each trial, unimodal (auditory, visual, or tactile), bimodal (audio-tactile, audio-visual, tactile-visual) or trimodal (audio-tactile-visual) stimuli with a duration of 100 ms were randomly produced by a randomized number of units in the array (between one and four active units), defined by 15 configurations. The active units produced the same temporally-congruent stimulation. Auditory stimuli were provided as 79 dB white noise burst at 300 Hz, visual stimuli were produced as white light by RGB LED (luminance: 317 mcd), and tactile stimuli were conveyed by a vibromotor peripheral (vibration frequency: 10 Hz). The experimental protocol was divided into two phases: (a) a perceptual phase, where participants were asked to tap the upper surface of the fifth unit with the right index finger as soon as they perceived a stimulus, regardless of the kind of stimulation conveyed; (b) cognitive phase, where they reported verbally how many devices they assumed as active. Each stimulation was reproduced three times in all configurations, with a total amount of 315 trials (45 trials per seven stimulation levels). The experiment was performed in about one hour, and short breaks were allowed at any time during the session.

### 2.3. Data Analysis and Statistics

The experiment was designed to evaluate: (a) the accuracy in determining the number of active units; (b) the responsiveness to different levels of stimulation on the body. As a measure of accuracy, we computed index correct (IC), calculated as the number of correct responses divided by the total number of trials for each configuration of stimuli, and expressed as an index between 0 and 1. As a measure of responsiveness, we collected reaction times (RT), calculated as the time interval between the beginning of the delivered stimulation and the time when the participant tapped the fifth unit with the right index finger and expressed in seconds (s). To evaluate whether data were normally distributed, we applied the Shapiro-Wilk test of normality with the free software R (Free Software Foundation, Boston, MA, USA). After verifying that the data did not follow a normal distribution, we ran the analysis using non-parametric statistics. We conducted two separate two-way permuted analyses of variance (ANOVAs) with IC and RT as dependent variables, and within-factors “stimulation” (seven levels: Auditory—A, Visual—V, Tactile—T, Audio-Tactile—AT, Audio-Visual—AV, Tactile-Visual—TV, and Audio-Tactile-Visual—ATV) and “active units” (four levels: One, Two, Three, and Four) as independent variables. The permuted Bonferroni correction for non-parametric data was applied in case of significant effects to adjust the *p*-value of multiple comparisons (significant value: *α =* 0.05).

## 3. Results

We carried out two levels of analysis: (i) the main effects of the increase of stimulated area and the types of stimulation provided on index correct (IC) and reaction times (RT); (ii) the interaction effects between increasing stimulated area and the kind of stimuli on IC and RT.

**Figure 2:**
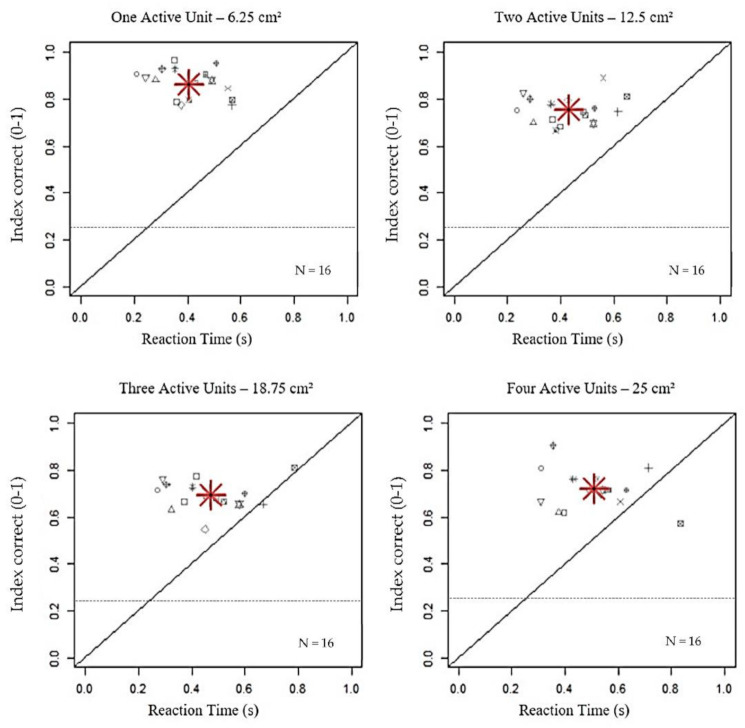
Impact of increasing stimulated area on index correct (IC) and reaction time (RT). The grey symbols represent the values of index correct (y-axis) between 0 and 1, as a function of Reaction Time (x-axis), expressed in seconds, per participant. The red asterisk is the average on the total number of participants. The black dashed lines indicate the chance level (0.25). The increase in the number of active units significantly resulted in a reduction of IC within 18.75 cm^2^ (*p* < 0.001), along with a linear increase of RT until 25 cm^2^ (*p* < 0.001).

In the first level of analysis, we examined whether the increasing number of active units and the difference between stimuli affected the performance in terms of accuracy (IC) and responsiveness (RT). As shown in Figure 2, the dimension of stimulated area resulted in a significant reduction of IC only within 18.75 cm^2^, with a similar performance in case of three and four active units, independently of the kind of stimulus provided (main effect: active units; Residual Sum of Squares (RSS) = 21.91, iter = 5000, *p* < 2.2 × 10^−16^). Differently from IC, reactions to stimuli linearly increased with the increasing number of sources (main effect: active units; RSS = 4.57, iter = 5000, *p* < 2.2 × 10^−16^). The presence of visual stimuli, alone (unimodal) or combined with auditory or/and tactile stimuli (bimodal, trimodal), increased the response correctness, while unimodal auditory, tactile and bimodal audio-tactile stimuli induced a lower accuracy, regardless of the stimulated surface (main effect: stimulation; RSS = 197.73, iter = 5000, *p* < 2.2 × 10^−16^). Concerning RTs, they were similar with unimodal tactile and bimodal visuo-tactile stimuli but higher than with other stimuli, apart from modality and number of active units (see Table 1 for Bonferroni corrections).

**Figure 3:**
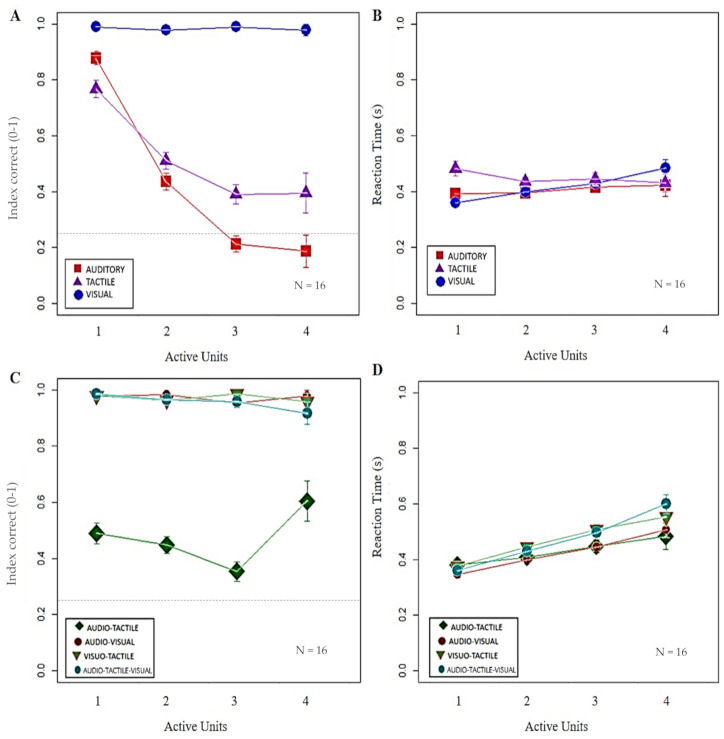
Impact of increasing stimulated area on index correct (IC) and reaction time (RT) with unimodal and multimodal stimuli. In (**A**,**B**) each symbol represents the mean value with standard error of IC and RT (y-axis), respectively, obtained by all the participants per number of active units (x-axis) with unimodal visual (blue dots), auditory (red squares) and tactile stimuli (purple triangles). The black dashed lines indicate the chance level (0.25). (**A**) Only visual stimuli induced a high IC (*p* = 1), regardless of the number of active units, with respect to the linear decrease of auditory (*p* < 0.05) and tactile (*p* < 0.01) modalities. (**B**) Concerning RT, a linear increase in responsiveness was present in case of visual stimuli (*p* < 0.01), but not auditory or tactile stimuli (*p* = 1). In (**C**,**D**) each symbol represents the mean value with standard error of IC and RT (y-axis), respectively, obtained by all the participants per number of active units (x-axis) with bimodal audio-tactile (green rectangle), audio-visual (dark-red dots), and visuo-tactile (red and green triangle) stimuli, and with trimodal audio-tactile-visual (light-blue dots) stimuli. (**C**) When bimodal conditions included visual stimuli, IC maintained a high value, independently from the number of active units (*p* = 1), while IC were lower for audio-tactile stimuli with no significant differences between different dimensions of stimulated areas (*p* > 0.10). (**D**) In opposition to the trend of IC, RT linearly delayed only in presence of visual stimuli (*p* < 0.05), while no significant difference was experienced with audio-tactile stimuli (*p* > 0.80).

The second level of analysis investigated whether the combination of the two factors might influence participants’ performance and responsiveness. Firstly, we compared unimodal stimuli by considering changes in IC and RT while increasing the stimulated area. Figure 3A shows that the number of correct responses remained high only in case of visual stimuli (interaction between stimulation x active units; RSS = 45.04, iter = 5000, *p* < 2.2 × 10^−16^), while it linearly decreased with the increase in the number of active units for auditory and tactile stimuli. By contrast, participants slowed down reactions to visual (inter action between stimulation x active units; RSS = 3.12, iter = 5000, *p* < 2.2 × 10^−16^) but not to auditory and tactile stimuli (see Figure 3B). Such result might be interpreted as a speed– accuracy trade-off. 

Secondly, we analyzed IC and RT’s trend in the case of bimodal and trimodal stimuli with increasing active units. As shown in the first level of analysis, Figure 3C highlights that vision combined with other stimuli improved the performance in terms of correctness, although a linear delay in responsiveness (see Figure 3D) was confirmed. Concerning audio-tactile stimuli, the absence of significant differences between different dimensions of stimulated area was observed for both IC and RT (see Table 1 for Bonferroni corrections).

**Figure 4:**
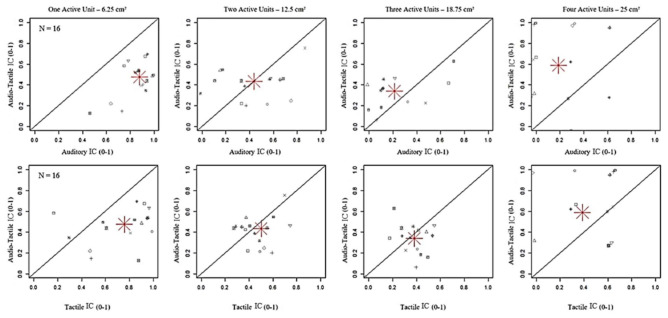
Comparison of index correct (IC) between bimodal Audio-Tactile stimuli and unimodal Auditory and Tactile stimuli. The grey symbols represent the values of index correct in the Audio-Tactile modality (y-axis), expressed as an index between 0 and 1, per participant. The red asterisk is the average on the total number of participants. IC was lower with bimodal than both unimodal stimulations when one unit was active (6.25 cm^2^) (*p* < 0.001), while it increased until 18.75 cm^2^ with a significant difference with respect to Auditory (*p* < 0.001) but not Tactile (*p* = 1.00) stimuli.

Moreover, to deeply evaluate the interaction between auditory and tactile stimuli, we compared the performance with unimodal and bimodal conditions (Figure 4). Results showed that IC was lower with bimodal than both unimodal stimulations for the smallest dimension of stimulated area (6.25 cm2). However, accuracy with audio-tactile stimuli surprisingly increased with the highest dimension of stimulated area (25 cm2), overtaking auditory stimulation, that was even lower than tactile stimulation with three active units (see Figure 3A), but not tactile stimulation (see Table 1 for Bonferroni corrections). These findings might point out that bimodal interaction of audio-tactile stimuli occurred with increased stimulation complexity.

Finally, we calculated the index of errors made by participants in case of auditory, tactile and audio-tactile stimulations, expressed as the number of times that participants attributed a wrong number of active units per condition divided by the total trials per condition. Errors were not randomly distributed across the three incorrect alternatives, but they were predominantly closed to the correct response, with a visible reduction of errors far from correct responses, e.g., when “4” is the correct response, there is a predominance of “3” together with “4”.

## 4. Discussion

The present study aims to unravel the mechanisms responsible for unisensory and multisensory spatial interaction, specifically investigating whether the stimulated area’s increase has a detrimental or beneficial effect on sensory threshold. Two main results came out from the present work, respectively related to the proposed task’s unisensory and multisensory conditions.

In terms of unisensory processing, we found that visual information dominates perception. This finding is in line with previous works demonstrating the higher reliability of vision in perceiving simultaneous stimuli [3,49,50,51] Research has also demonstrated that the combination of perceptual experiences from the environment and visual experience drives the development of allocentric spatial skills [52,53]. Moreover, spatial accuracy and precision of an event improve when multiple senses, congruent in space and in time, are integrated [16]. However, our findings showed that when the stimulated area increases, a delayed responsiveness can be observed in the visual domain, indicating that size of stimulation has an effect in terms or visual responsiveness. A possible explanation might be that, when stimulation is conveyed on the body, the faster response of touch is due to the fact that touch contributes to represent space based on egocentric (bodily) coordinates. Concerning hearing, we found that auditory responsiveness was not affected by the size of the stimulated area but auditory accuracy did not improve along with the increase of stimulated area. This might be due to the fact that auditory information is less reliable than visual information based on external landmarks and less reliable than tactile information on the body. Several studies have demonstrated that vision typically dominates other sensory modalities in perception, producing a strong bias in case of conflicting events [22,23,24,25,26,27]. Our result might suggest that increasing the surface area on the body produced a sensory conflict between multiple sensory modalities, independent of the spatial and temporal coherence of stimulation.

Consequently, we might argue that conflicting events are solved by vision under a more cognitive point of view, while auditory and tactile stimuli foster perceptual abilities when multiple and proximal stimuli add up in space. A further explanation of the late responsiveness of vision in the task proposed might be related to the coexistence of retinotopic and spatiotopic reference frames to build spatial maps of the environment [54,55,56,57]. Since retinotopic coordinates can induce an error signal when the fovea has to be moved and kept on a selected target, such a reference system can be considered more viewer-centered than spatiotopic frames of reference determine observer-independent properties of stimuli [58]. According to this view, we might conclude that retinotopic and spatiotopic frames of reference come into a conflict when perceptual features are processed, producing a significant delay in responsiveness, while spatiotopic coordinates overcome when cognitive processes take place and guide the other senses in encoding bodily space.

In terms of multisensory processing, we found that audio-tactile interaction enhances sensory accuracy more than unisensory (audio, tactile) processing only when the stimulated area increases. Indeed our results indicate that a significant increase in sensory accuracy is evident only when the surface areas correspond to four devices (25 cm^2^), suggesting that the dimension of stimulated area might facilitate multisensory interaction. A possible reason for this is that, while vision dominates the spatial representation, a cost for integrating bigger spatial and tactile areas might emerge. Vision might work as a glue between spatial coordinates of the different senses. When vision is not available, the association between auditory and tactile stimuli might have a stronger benefit for a bigger area of stimulation, where unisensory uncertainty is smaller. This idea might be supported by previous findings on the computation of frequency that showed a convergence between auditory and tactile stimuli in the case of spatiotemporal coherence [59]. According to such a view, other works have highlighted the early convergence and integration of auditory and tactile inputs at sensory cortices level [60,61,62,63,64,65,66]. When vision is not available, we may suppose that touch plays a pivotal role in audio–tactile interaction since it processes spatial information conveyed on the body based on body-centered coordinates. In this sense, touch might be considered a more reliable sensory cue when multiple stimuli are conveyed in proximal positions and simultaneously. The dominance of touch over audition has been reported in previous works on spatiotemporal information processing within peripersonal borders, even though it might depend on body posture changes [67,68]. This concept is also shown in several studies on audio–tactile interaction, demonstrating that the presence of tactile stimuli seems to impact auditory perceptual judgments more than auditory information on tactile judgments [67,69,70].

## 5. Conclusions

The present work aimed to unravel the role of vision combined with audition and touch in space representation in an increasing bodily area. Our results suggested that, since multisensory interaction is driven by vision improved response accuracy with the increasing of the stimulated area, vision provides a more reliable information to encode peripersonal space, namely object-centered or allocentric. This result supports the idea that allocentric frames of reference (vision) enhances spatial change discrimination when multiple stimuli occur in the same location and time. On the other hand, relying on visual cues might affect responsiveness to stimulation. Depending on vision seems to have a cost in terms of the spatial perception of both unisensory and multisensory events, while touch might guide audition in the discrimination of an increasing audio-tactile area on the body. Indeed, the combination of audition and touch improved the performance compared to unimodal auditory stimuli for a high number of active units, which might highlight a leading role for touch, based on body-centered spatial coordinates, in an audio–tactile interaction process. These findings would indicate the value of further investigation on the relevance of spatial and temporal coherence when multisensory stimulation sources add up in space during different developmental stages from childhood, considering also the combination of peripersonal and extrapersonal stimuli and how extrapersonal space may impact on spatial processing of multisensory contingencies.

## Figures and Tables

**Table 1:** Results with Bonferroni corrections. The table reports the results of the main effects and interaction effects for the index correct (IC) and responsiveness (RT).

**Main Effects**	**Bonferroni-IC**	**Bonferroni-RT**
Active Units	One vs. Two: *p* < 0.001	One vs. Two: *p* = 0.02
	One vs. Three: *p* < 0.001	One vs. Three: *p* < 0.001
	One vs. Four: *p* < 0.001	One vs. Four: *p* < 0.001
	Two vs. Three: *p* < 0.001	Two vs. Three: *p* < 0.001
	Two vs. Four: *p* = 0.89	Two vs. Four: *p* < 0.001
	Three vs. Four: *p* = 1.00	Three vs. Four: *p* = 0.008
Stimulation		T vs. TV: *p* = 1.00
		T vs. A: *p* = 0.017
	V vs. A/T/AT: *p* < 0.001	T vs. V: *p* = 0.004
	AV vs. A/T/AT: *p* < 0.001	T vs. AT: *p* = 0.025
	TV vs. A/T/AT: *p* < 0.001	T vs. AV: *p* < 0.001
	ATV vs. A/T/AT: *p* < 0.001	T vs. ATV: *p* < 0.001
		TV vs. A: *p* = 0.004
		TV vs. V/AT/AV: *p* < 0.001
**Interaction Effects**	**Bonferroni-IC**	**Bonferroni-RT**
Stimulation × Active Units	V-One vs. V-One/Two/Three/Four: *p* = 1.00	V-One vs. V-Three/Four: *p* < 0.001
	A-One vs. A-Two/Three/Four: *p* < 0.001	V-Two vs. V-Four: *p* = 0.004
	A-Two vs. A-Three: *p* = 0.049	A-One vs. A-One/Two/Three/Four: *p* = 1.00
	T-One vs. T-Two/Three/Four: *p* < 0.001	T-One vs. T-One/Two/Three/Four: *p* = 1.00
	T-Two vs. T-Three: *p* < 0.001	AV-One vs. AV-Two: *p* = 0.005
	T-Two vs. T-Four: *p* = 0.007	AV-One vs. AV-Three/Four: *p* < 0.001
	T-Three vs. A-Three: *p* = 0.038	
	AV/TV/ATV-One vs. AV/TV/ATV-Two/Three/Four: *p* = 1.00	AV-Three vs. AV-Four: *p* = 0.037
	AT-One vs. AT-Two/Four: *p* = 1.00	TV-One vs. TV-Two: *p* = 0.024
	AT-One vs. AT-Three: *p* = 0.72	ATV-One/Two vs. ATV-Three/Four: *p* < 0.001
	AT-Two vs. AT-Three/Four: *p* = 1.00	ATV-Three vs. ATV-Four: *p* = 0.026
	AT-Three vs. AT- Four: *p* = 0.19	AT-One vs. AT-Two: *p* = 1.00
	AT-One vs. A-One: *p* < 0.001	AT-One vs. AT-Three: *p* = 0.88
	AT-One vs. T-One: *p* < 0.001	AT-One vs. AT-Four: *p* = 0.96
	AT-Four vs. A-Four: *p* < 0.001	AT-Two vs. AT-Three/Four: *p* = 1
	AT-Four vs. T-Four: *p* = 1.00	AT-Three vs. AT-Four: *p* = 1.00

## Data Availability

The data presented in this study are available on request from the corresponding author. The data are not publicly available due to privacy.
